# The Association of Meal Timing With Body Composition and Cardiometabolic Health in Obese Older Adults

**DOI:** 10.1093/geroni/igab046.200

**Published:** 2021-12-17

**Authors:** Samaneh Farsijani, Nancy W Glynn, Anne Newman

**Affiliations:** 1 University of Pittsburgh, Pittsburgh, Pennsylvania, United States; 2 University of Pittsburgh Graduate School of Public Health, Pittsburgh, Pennsylvania, United States

## Abstract

Objectives: To determine the association between eating window and time of last calorie intake with body composition and cardiometabolic health in obese older adults. Methods: We performed a cross-sectional analysis on 36 community-dwelling, overweight-to-obese (BMI 28.0-39.9 kg/m2) older adults, recruited to participate in a weight loss and exercise trial. Time of food intake were extracted from three 24-hour food recalls. Eating window was calculated as the time elapsed between the first and last food intake. We recorded the time of last calorie intake either from food or drink. Blood glucose, triglycerides, high-density (HDL) & low-density (LDL) lipoprotein cholesterols were measured as markers of cardiometabolic health. Total fat and lean mass were assessed by DXA. Partial correlation was used to determine the relationships between eating window and last calorie intake with body composition and cardiometabolic markers, while controlling for sex, age, and total calorie intake. Results: On average, participants’ eating window was 12.0±1.1 hours. Time of last calorie intake in 86% of participants was between 6:00-8:00 PM. After controlling for potential confounders, longer eating windows were associated with higher triglyceride levels (P=0.032) and lower HDL (P=0.035), while no association was observed with the other cardiometabolic markers. We observed negative trends, though not statistically significant, between longer eating windows and greater weight, BMI, and fat mass. No association was observed between time of last calorie intake, body composition and cardiometabolic markers. Conclusions: Our results suggest that timing of food intake may influence cardiometabolic risk and obesity in older adults.

